# Magnetic resonance imaging findings in a patient with seropositive neuromyelitis optica

**DOI:** 10.4102/sajr.v22i1.1306

**Published:** 2018-08-30

**Authors:** Siviwe S. Mpateni, Naye C. Sihlali, Emma C. Gardiner, Nkululo Gigi

**Affiliations:** 1 Department of Diagnostic Radiology, Livingstone Tertiary Hospital, Port Elizabeth, South Africa; 2 Rheumatology Department, Livingstone Tertiary Hospital, Port Elizabeth, South Africa; 3 Department of Internal Medicine, Livingstone Hospital, Port Elizabeth, South Africa

## Abstract

We present the case of a 23-year-old female with a subacute history of complex additive neurology which consisted of progressive unilateral visual impairment and subsequent blindness of the right eye, in conjunction with distal lower motor neuron symptoms of weakness and sensory loss from T4 level down. Special investigations performed, included serology and an urgent magnetic resonance imaging (MRI) of the brain and spinal cord, which exhibited a diffuse demyelinating disease of the brain and spinal cord without the typical features of multiple sclerosis (MS) and laboratory findings, which were positive for the AQP-4 antibody, confirming the diagnosis of neuromyelitis optica (NMO). Pulsed methylprednisolone was initiated urgently with good effect and immunosuppression with cyclophosphamide was added after the exclusion of additional pathology. She experienced a complete resolution of her weakness and sensory impairment upon discharge; however, her unilateral visual loss remained. The recent advances in the identification of autoimmune biomarkers and the widening spectrum of imaging findings in NMO necessitate that the clinician and radiologist keep abreast of the current diagnostic tools and criteria that distinguish NMO from other demyelinating conditions.

## Introduction

Neuromyelitis optica (NMO), also known as Devic’s disease, is an idiopathic demyelinating disease of the central nervous system that is typified by severe and intermittent involvement of the optic nerve, spinal cord and brain. This clinical entity was previously considered a variant of multiple sclerosis (MS), but recent developments in clinical, radiological and immunological characteristics of the disease have paved the way for a more detailed definition of this condition as a separate neurological affliction.^1,2,3^

The aetiology of this condition is driven by the action of the autoantibody, namely, NMO-immunoglobulin G (IgG), against aquaporin-4 (AQP4) water channels. Aquaporin-4 channels are highly populated in the astrocytes which facilitate the transportation of water through cell membranes.^4,5^ The spinal cord and optic nerves are occupied by aquaporin channels and appear to be the preferential targets, probably because of the increased permeability of the blood-brain barrier in these regions.^4^

The previous classification of NMO regarded the condition as a clinical subtype or variant of MS, and this resulted in delayed detection and misrecognition of NMO. The early differentiation between NMO and MS is particularly important because the course of NMO is more severe and the treatment strategies for attack prevention differ. Immunotherapies approved for the treatment of MS have proven ineffective and even appear to aggravate NMO relapses.^1^

The diagnostic limitations of complex neurological findings in patients who present with overlapping upper and lower motor neuron signs remain a challenge for health care practitioners, especially in resource-limited centres. The cohesive application of clinical, radiological and immunological findings to reach a definitive diagnosis is of the utmost importance in such settings.

## Case report

We present the case of a 23-year-old female who was referred to Livingstone Tertiary Hospital with a 1-year history of Coombs-positive autoimmune haemolytic anaemia secondary to Systemic Lupus Erythematosus (SLE). She was previously on corticosteroid therapy which was discontinued and she had no other established organ involvement.

She developed progressive loss of vision in the right eye, in association with left hemiparesis and subtle sensory deficit extending inferiorly from the level of T4. On clinical examination, she was fully conscious and oriented; her vision was limited to light perception in the right eye and fundoscopy revealed a clinically pale right optic disc.

Further neurological assessment revealed that she had motor weakness with 4-/5 power in the left upper and lower limbs and sensory impairment predominantly affecting the left side with a T4 sensory level. In addition, she had bilateral sensorimotor weakness superimposed on her left hemiparesis and features of chronic distal lower motor neuron polyneuropathy.

**FIGURE 1:**
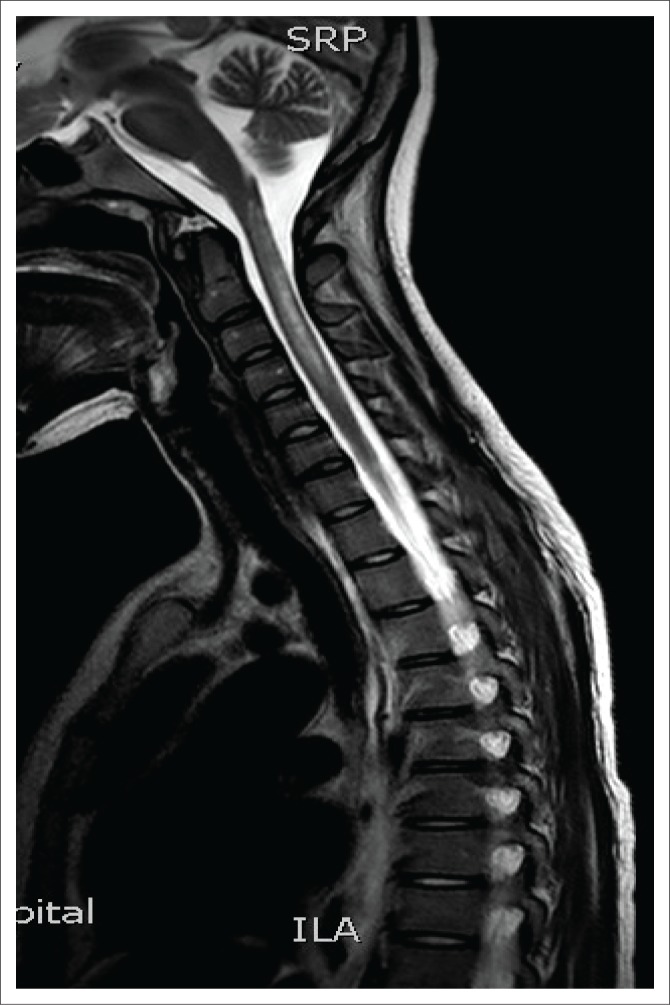
Sagittal T2-weighted magnetic resonance image depicting a hyperintense longitudinally extensive spinal cord lesion extending from the cervicomedullary junction to the C4 vertebra.

**FIGURE 2:**
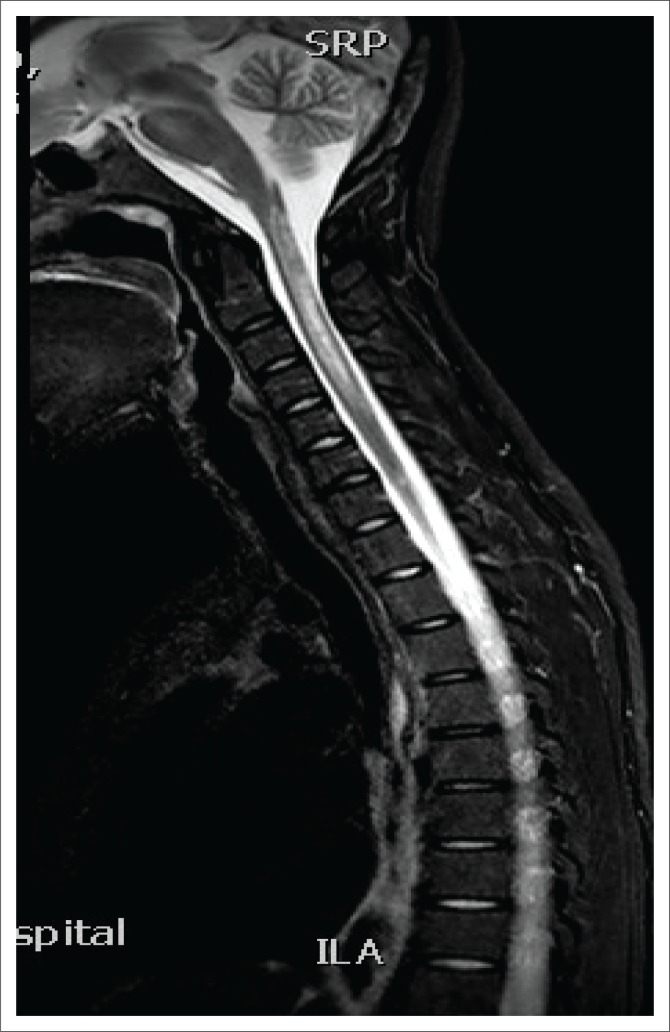
Sagittal short TI inversion recovery magnetic resonance images demonstrating longitudinally extensive spinal cord lesion.

**FIGURE 3:**
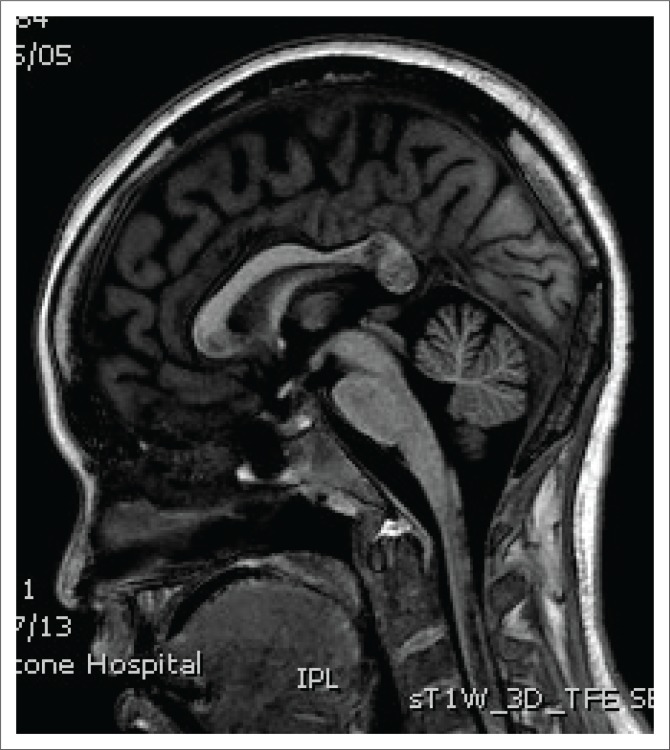
Sagittal T1-weighted image depicting hypointensity in the splenium and cervical spinal cord.

**FIGURE 4:**
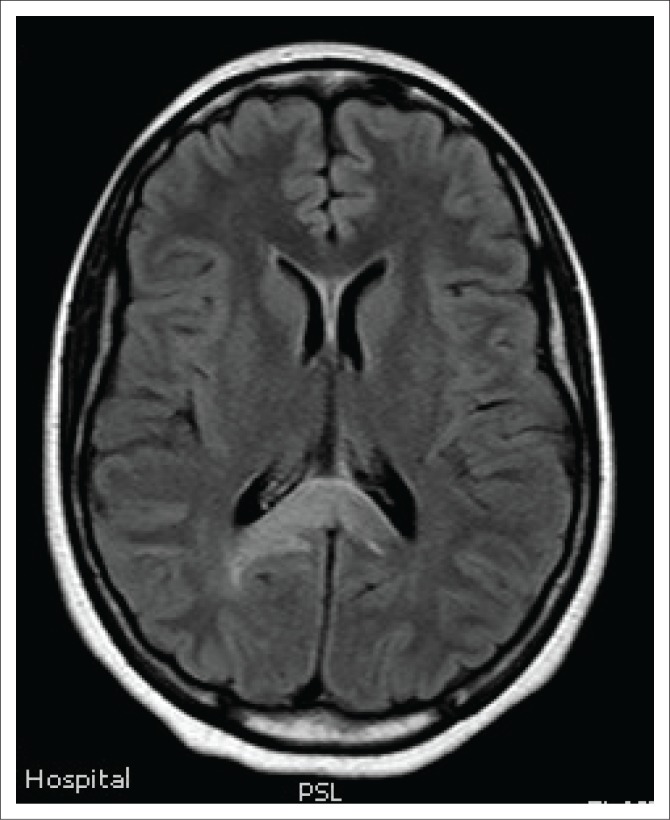
Axial fluid-attenuated inversion recovery magnetic resonance image demonstrating hyperintensity in the splenium of corpus callosum in a ‘bridging arch pattern’.

**FIGURE 5:**
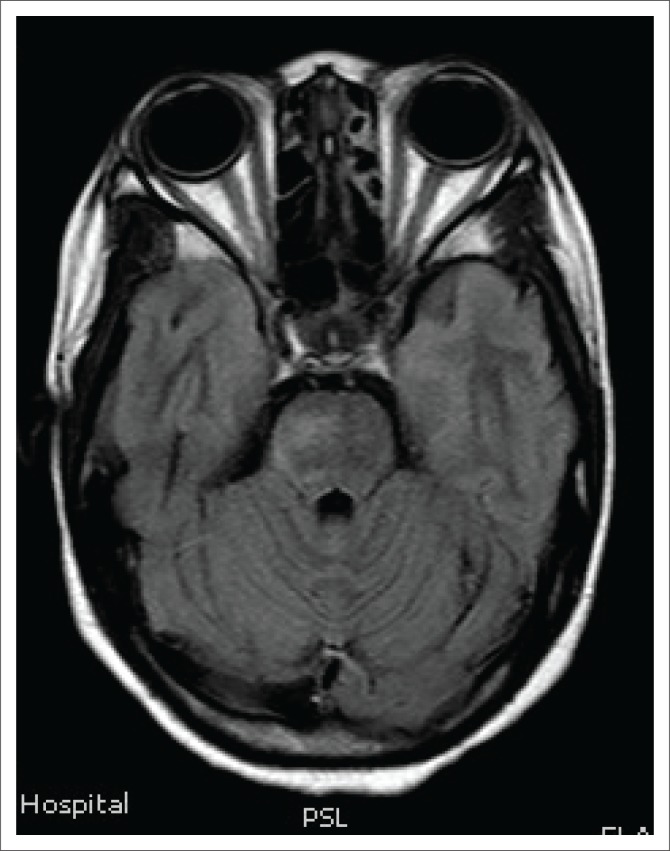
Axial fluid-attenuated inversion recovery magnetic resonance showing patchy hyperintensity in the midbrain and depicting faint hyperintensity of the right posterior optic nerve.

**FIGURE 6:**
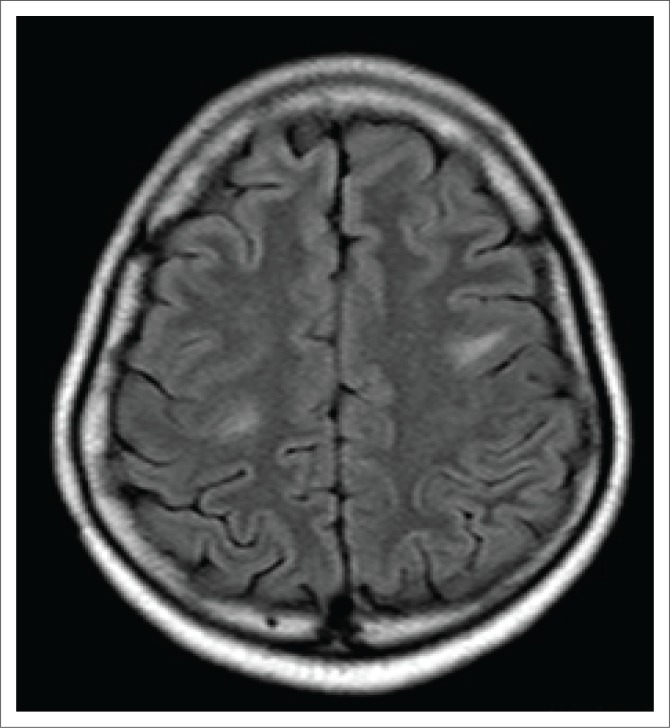
Axial T2-weighted image with hyperintense subcortical white matter lesions.

Initial investigations included serology and urgent magnetic resonance imaging (MRI) of the brain and spinal cord with contrast (Figures 1–6).

Magnetic resonance imaging of the cervical spine revealed a T2 hyperintense central grey matter lesion extending longitudinally and contiguously across three vertebral body lengths. Brain MRI demonstrated similar T2 hyperintense lesions involving the splenium of the corpus callosum, the midbrain and centrum semiovale. There were no perivenular radially oriented lesions (Dawson fingers) that are typical of MS.

Cerebrospinal fluid analysis demonstrated no lymphocytes, normal protein and negative oligoclonal bands. Serum studies were positive for AQP4-antibodies (NMO-IgG), confirming the diagnosis of NMO. Treatment with high-dose corticosteroid therapy and cyclophosphamide was initiated and this led to a complete resolution of the patient’s weakness and sensory deficit; however, the visual impairment in the right eye persisted.

## Discussion

There are limited studies on the prevalence of NMO in the Southern African population, especially in human immunodeficiency virus-(HIV) negative subjects, and this condition is likely underdiagnosed. North American studies depict ranges from 0.5 to 4.4 per 100 000, with a higher incidence in Asian populations, Indian populations and African populations, with women being more commonly affected with a ratio of 6.5–1.^4^

Acute attacks are characterised by progressive loss of vision in one or both eyes, varying degrees of weakness or paralysis in the legs or arms, sensory deficits as well as bladder and/or bowel dysfunction. The onset of optic neuritis can be unilateral or bilateral and may be confounded by concurrent myelitis; hence, serological findings can serve to be a valuable diagnostic tool. NMO-immunoglobulin G offers a specificity of more than 90% and sensitivity of 70% – 90% for this condition.^5^

Conventional magnetic resonance (MR) imaging changes have traditionally been recognised in the spinal cord and optic nerves. However, recent studies documenting cerebral involvement have mandated the inclusion of cerebral involvement as one of the major supportive criteria.

The lesions in NMO are typically hyperintense on T2-weighted images. The distribution is observed in the regions of the central nervous system known to be rich in AQP4, typically in a periependymal distribution in the periventricular white matter, corpus callosum and central grey matter of the spinal cord.^5^

Both MS and NMO present with circumventricular cerebral lesions; however, there are salient defining imaging features that distinguish these conditions on imaging. MS frequently presents with discrete, ovoid lesions which lie perpendicular to the ventricles (radially oriented perivenular lesions commonly referred to as ‘Dawson fingers’).^5,6^ In contradistinction, the lesions in NMO occur along the ependymal lining of the lateral ventricles, the regions around the third ventricle, cerebral aqueduct and fourth ventricle. These are heterogeneous, oedematous and form large confluent lesions creating a ‘marbled pattern’. There may be notable involvement of the splenium presenting as a unique ‘bridging arch pattern’ as was demonstrated in this case (Figure 4).^2,5,6^ Similar large, confluent and heterogeneous lesions occur in the cerebral white matter, which may be contiguous with the periependymal lesions.

Spinal cord imaging features in NMO demonstrate the presence of longitudinally extensive spinal cord lesions (LESCLs), which extend contiguously across at least three vertebral segments.^2,3,4^ LESCLs are, however, not exclusive to NMO and appear to be less disease specific in children than in adults. They are frequently observed in children with acute disseminated encephalomyelitis, as well as in 17% of those with MS and in 67% – 88% of those with monophasic transverse myelitis.^5,6^ In NMO there is a preferential involvement of the central grey matter which is abundant with AQP4 in the glial cell processes adjacent to the ependymal cells of the central canal with lesions frequently affecting the cervical and thoracic spinal cord.^3,5^ Lesions in NMO exhibit high signal intensity on T2-weighted images and low signal intensity on T1-weighted images.

In MS, however, hypointense lesions on T1-weighted images are very rare. The typical spinal cord features of MS are of multiple, short lesions which have a peripheral, asymmetric and often posterior distribution.^6^

Optic nerve changes occur in both NMO and MS. The associated imaging features of both depict acute nerve sheath thickening and enhancement; however, long-segment inflammation that extends posteriorly to the optic chiasm should rouse the suspicion of NMO, especially when findings are simultaneous and bilateral.^5,6^

The current diagnostic criteria for NMO mandate the occurrence of: (1) optic neuritis, (2) myelitis and (3) at least two of the following three supportive criteria:

- longitudinally extensive spinal cord lesions
- seropositivity for IgG antibodies that bind AQP4 on astrocytes
- brain imaging findings non-diagnostic for MS.^2,5^

The fulfilment of the current diagnostic criteria differentiates NMO from other demyelinating disorders, which is crucial as specific therapeutic interventions are required to reduce the frequency of flare-ups. MR imaging in patients with NMO has revealed an expanding array of lesion subtypes with which the radiologist must be familiar.^5^ In presenting this case, we would like to draw special attention to the imaging features and the diagnostic biomarkers that assist in defining NMO as a clinical entity.

## Conclusion

Novel immunological investigations (NMO-IgG/AQP4-antibody) have had a tremendous impact in achieving greater diagnostic accuracy. With this case we hope to highlight the diagnostic complexity encountered when analysing the imaging findings in NMO and encourage a high-index of suspicion when met with the above-mentioned imaging features.
